# Application of Electroencephalography (EEG) in Combat Sports—Review of Findings, Perspectives, and Limitations

**DOI:** 10.3390/jcm14124113

**Published:** 2025-06-10

**Authors:** James Chmiel, Jarosław Nadobnik

**Affiliations:** Faculty of Physical Culture and Health, Institute of Physical Culture Sciences, University of Szczecin, Al. Piastów 40B blok 6, 71-065 Szczecin, Poland

**Keywords:** combat sport, EEG, electroencephalography, sport, electroencephalogram, brain oscillations, QEEG, sport performance, neurophysiology, neural correlates

## Abstract

**Introduction:** Combat sport athletes are exposed to repetitive head impacts yet also develop distinct performance-related brain adaptations. Electroencephalography (EEG) provides millisecond-level insight into both processes; however, findings are dispersed across decades of heterogeneous studies. This mechanistic review consolidates and interprets EEG evidence to elucidate how participation in combat sports shapes brain function and to identify research gaps that impede clinical translation. **Methods:** A structured search was conducted in March 2025 across PubMed/MEDLINE, Scopus, Cochrane Library, ResearchGate, Google Scholar, and related databases for English-language clinical studies published between January 1980 and March 2025. Eligible studies recorded raw resting or task-related EEG in athletes engaged in boxing, wrestling, judo, karate, taekwondo, kickboxing, or mixed martial arts. Titles, abstracts, and full texts were independently screened by two reviewers. Twenty-three studies, encompassing approximately 650 combat sport athletes and 430 controls, met the inclusion criteria and were included in the qualitative synthesis. **Results:** Early visual EEG and perfusion studies linked prolonged competitive exposure in professional boxers to focal hypoperfusion and low-frequency slowing. More recent quantitative studies refined these findings: across boxing, wrestling, and kickboxing cohorts, chronic participation was associated with reduced alpha and theta power, excess slow-wave activity, and disrupted small-world network topology—alterations that often preceded cognitive or structural impairments. In contrast, elite athletes in karate, fencing, and kickboxing consistently demonstrated neural efficiency patterns, including elevated resting alpha power, reduced task-related event-related desynchronization (ERD), and streamlined cortico-muscular coupling during cognitive and motor tasks. Acute bouts elicited transient increases in frontal–occipital delta and high beta power proportional to head impact count and cortisol elevation, while brief judo chokes triggered short-lived slow-wave bursts without lasting dysfunction. Methodological heterogeneity—including variations in channel count (1 to 64), reference schemes, and frequency band definitions—limited cross-study comparability. **Conclusions:** EEG effectively captures both the adverse effects of repetitive head trauma and the cortical adaptations associated with high-level combat sport training, underscoring its potential as a rapid, portable tool for brain monitoring. Standardizing acquisition protocols, integrating EEG into longitudinal multimodal studies, and establishing sex- and age-specific normative data are essential for translating these insights into practical applications in concussion management, performance monitoring, and regulatory policy.

## 1. Introduction

Combat sports—boxing, wrestling, judo, taekwondo, mixed martial arts (MMA), etc.—feature regulated grappling and/or striking and are classed as grappling, striking, or mixed. They attract millions of fans worldwide and make up ~26% of Summer Olympic medals (across boxing, judo, and taekwondo) [1]. Divisions are defined by weight; to enter lighter classes, many athletes use rapid weight loss (RWL), an acute strategy that, when excessive, impairs hydration and cognition [2,3,4]. Physically, bouts comprise 1–5 min high-intensity rounds separated by brief rests, demanding both anaerobic power and aerobic capacity [5]. MMA couples explosive exchanges with short recoveries, so fighters require high peak anaerobic output and moderate-to-high VO_2_ max [6,7]. Striking sports (such as boxing and taekwondo) emphasize speed and power, whereas grappling sports (such as wrestling and judo) rely on maximal strength over longer efforts [8]. Mixed disciplines therefore call for a broad profile—strength, speed, power, endurance, agility, and flexibility—and elite athletes typically display lean, mesomorphic builds blending these qualities [9,10]. Psychological attributes are equally decisive: athletes must stay focused, confident, and emotionally controlled, making split-second decisions while managing fear and aggression. Mental toughness, self-confidence, situational awareness, and resilience differentiate elite from novice fighters, alongside superior attention, reaction time, and stress tolerance [11]. Because of these stresses, coaches monitor internal load via heart rate, blood lactate, and rating of perceived exertion (RPE); RPE correlates strongly with lactate (r ≈ 0.8) and with training impulse metrics such as Edwards’ and Banister’s TRIMP [12]. Accurate load tracking is essential as combat sports impose extreme and intermittent technical and tactical efforts.

Head impacts pose the greatest injury risk. In MMA, 88.1% of knockouts result from head blows, rising to 54% of all bouts ending in knockouts in heavier divisions [13]; boxing reports 74–96% head injuries with concussions comprising up to 75% [14], and wrestling registers 269.3 mild traumatic brain injuries per 100,000 person-years—far exceeding boxing (85.6) and other martial arts (61.0) [15]. Repeated trauma can lead to chronic traumatic encephalopathy (CTE)—a progressive disease marked by cognitive decline, mood disturbance, and dementia [16,17]. CTE has been confirmed in several MMA fighters and numerous boxers [16]; up to 40% of retired boxers show chronic brain injury symptoms [14], and contact sport athletes—including football players—constitute ~86% of diagnosed CTE cases [18].

Because combat sports expose competitors to frequent head trauma and its associated risks, there is a clear need to monitor how participation in contact sports affects brain function. Electroencephalography (EEG)—the oldest and one of the most widely used methods for recording the bioelectrical activity of the brain—provides a valuable tool for this purpose.

EEG is a non-invasive technique used to record the electrical activity of the brain [19]. It operates by placing electrodes on the scalp to detect voltage fluctuations resulting from the synchronized firing of neurons, particularly the pyramidal cells in the cerebral cortex [20]. When large populations of these neurons communicate through electrical impulses, and when large groups of them fire simultaneously, their collective electrical signals can be measured at the scalp surface [21]. These signals—called brainwaves—follow rhythmic patterns of neural activity and are grouped into frequency bands: delta (0.5–4 Hz), theta (4–8 Hz), alpha (8–13 Hz), beta (13–30 Hz), and gamma (>30 Hz). Each band corresponds to a general brain state: delta is linked to deep sleep [22], theta to light sleep [23], alpha to relaxed wakefulness [24], beta to active thought and concentration [25], and gamma to complex cognitive processing [26]. EEG provides extremely high temporal resolution—on the order of milliseconds—which makes it ideal for monitoring fast neural processes [27]. It is frequently used in research to investigate sensory processing, language processing, attention, decision-making, and other cognitive functions. Unlike imaging techniques such as MRI or CT scans, EEG does not provide high spatial resolution, meaning it cannot localize the exact source of neural activity deep within the brain [28]. However, its speed and safety make it an excellent tool for tracking changes in brain activity over time. Clinically, it is used for diagnosing neurological conditions such as epilepsy [29], sleep disorders [30], brain injuries [31], and encephalopathies [32]. It can detect abnormal patterns of brain activity, such as epileptic spikes or seizure activity, and is often employed during sleep studies or in intensive care units.

In sports science, EEG has become increasingly useful for studying the neural mechanisms that underlie athletic performance. This technique has been applied across sports disciplines to explore cognitive processing, motor control, training effects, and even injury assessment. One of the primary limitations of EEG in sports is the difficulty of obtaining clean data during physical activity. Movement introduces muscle artifacts and electrode displacement, which compromise data quality. To address this, researchers often record EEG during before activity, during simulations, or even through imagined motor tasks [33]. These methods allow researchers to collect valid data while limiting motion-related artifacts. In target-based sports such as archery, golf, and rifle shooting, EEG recordings before performance have shown that successful attempts are often preceded by distinct neural patterns, especially alpha desynchronization in motor-related brain areas [33].

The neural efficiency hypothesis suggests that expert athletes exhibit lower cortical activation when performing tasks at a high level, implying more efficient processing. EEG studies support this idea, showing that elite athletes have reduced alpha event-related desynchronization (ERD) and increased event-related synchronization (ERS) compared to novices, especially during motor preparation and decision-making [34,35]. Frequency-specific analyses have linked distinct oscillatory patterns to cognitive and motor performance, where enhanced theta activity is associated with increased attentional engagement, especially in the frontal lobe, while beta activity often reflects motor readiness. For example, elite badminton and football players show greater frontal theta and parietal alpha ERD, which correlates with quicker response times and better spatial accuracy [34].

New technologies allow EEG to be recorded during stationary physical activities like cycling. These studies show that physical exertion increases cortical arousal—for example, moderate exercise has been linked to increased P300 amplitude in ERP studies, indicating improved cognitive processing during or shortly after exertion [36]. Long-term physical activity is also associated with improved ERP components and attentional control, reflecting the cognitive benefits of aerobic fitness [36]. However, standard scalp EEG is not feasible during live combat. First, the vigorous movements involved generate severe motion and muscle artifacts, making the recordings unusable. Second, because electrodes are attached to the head, repeated blows risk dislodging or damaging both the sensors and the recording hardware.

Simulated sports settings and motor imagery are also used to study neural activity without actual movement [37]. Imagined movements activate similar patterns to real actions, particularly in the alpha and mu bands [37]. This approach not only overcomes motion artifacts but also provides a foundation for brain–computer interface (BCI) applications in sports training [33]. Beyond performance monitoring, EEG is utilized to assess the neurological impact of head trauma [38]. Changes in EEG patterns in football suggest its usefulness in concussion protocols and long-term brain health surveillance [33]. Additionally, neurofeedback techniques—which allow athletes to train their brain activity using EEG signals—are also being tested for improving focus and performance under pressure [33].

The aim of this mechanistic review is to collect and evaluate studies that use EEG to examine brain activity in combat sports athletes. Although many neuroimaging methods are used in sports research, EEG remains one of the most established. It is non-invasive, portable, and cost-effective, making it suitable for use in a variety of settings. Given the risk of head trauma in combat sports, EEG provides a valuable way to study how training, competition, and skill level influence brain activity. Understanding the abnormalities and deviations in EEG patterns could also support the development of therapeutic strategies—such as non-invasive brain stimulation, computer–brain interfaces, and neurofeedback—to reduce the negative effects of participation, enhance performance, and monitor the neurological state of athletes. Only studies using raw EEG data recorded at rest or during tasks were included in this review. Studies that relied on event-related potentials (ERPs) or similar methods were excluded to ensure methodological consistency. Only publications from 1980 onward were considered, as older studies may not meet current standards of EEG recording and analysis.

## 2. Methods

This mechanistic review aims to explore how EEG is used to understand brain mechanisms in combat sports athletes. Although it draws on some principles of systematic reviews—such as structured search and study selection—its primary goal is not to assess effectiveness or outcomes but to explain the underlying brain processes identified through EEG. Given its mechanistic focus, this review does not follow the full PRISMA protocol typically required for systematic reviews. It also omits formal assessments like risk of bias or the PICOS framework.

### 2.1. Data Sources and Search Strategy

To identify relevant studies, two researchers (J.Ch. and J.N.) independently conducted a comprehensive literature search using a combination of keywords related to EEG and combat sports. The search terms included “EEG”, “QEEG”, “electroencephalogram”, “electrophysiology”, paired with “combat sport”, “MMA”, “boxing”, “judo”, “karate”, “jiu-jitsu”, “kickboxing”, and “K-1”, among others. The search was carried out in March 2025 and included multiple databases—PubMed/Medline, Scopus, ResearchGate, Google Scholar, and Cochrane—focusing on studies published from January 1980 to March 2025. In addition, the Google browser was also used, and similar and cited articles were searched in the PubMed database.

### 2.2. Study Selection Criteria

To be eligible for inclusion, studies had to be clinical trials published in English within the specified timeframe and specifically focused on EEG activity in combat sports athletes. Review papers, case reports, and studies in languages other than English were excluded.

### 2.3. Screening Process

A multi-step screening process was used to select studies that met the inclusion criteria. First, the titles and abstracts of all retrieved studies were reviewed independently by both researchers for relevance.

#### 2.3.1. Title and Abstract Screening

Each reviewer independently evaluated the titles and abstracts to identify papers that discussed EEG use in combat sports athletes. Only those aligned with the topic were considered for the next stage.

#### 2.3.2. Full-Text Assessment

Articles that passed the initial screening were then assessed in full to confirm that they met all eligibility criteria, particularly that they were clinical trials involving combat sports athletes, published in English between January 1980 and March 2025.

## 3. Results

**Figure 1:**
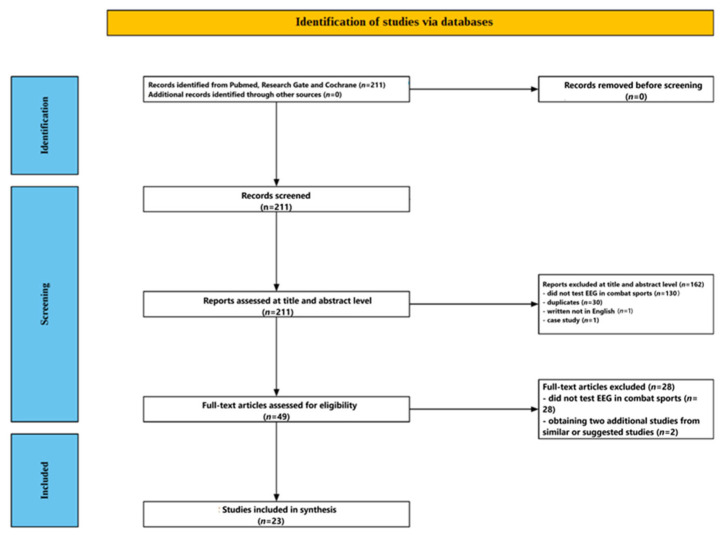
Flow chart depicting the different phases of the systematic review.

Figure 1 shows the flow of the screening process. The initial database search yielded 211 studies. After reviewing the titles and abstracts, 162 papers were excluded—130 for being unrelated to EEG in combat sports, 30 as duplicates, 1 for being written in a language other than English, and 1 for being a case study. The remaining 49 articles underwent full-text review, during which 28 were excluded for not directly addressing EEG in combat sport contexts. Two additional relevant studies were identified through PubMed’s related articles function. In total, 23 studies met all inclusion criteria and were selected for the final analysis.

### 3.1. Participant Characteristics

The included studies are presented in Table 1. The 23 studies [39,40,41,42,43,44,45,46,47,48,49,50,51,52,53,54,55,56,57,58,59,60,61] that fulfilled the inclusion criteria collectively involved approximately 650 combat sport athletes and 430 control participants. Athletes represented seven disciplines—boxing [39,40,41,42,43,44], kickboxing [48,49,59,60,61], karate [51,52,53,54,55,56,57,58], judo [39,50], wrestling [47], fencing, and mixed judo–boxing samples [55,56,57]—spanning a wide competitive range from novice amateurs with fewer than 5 bouts to professionals with over 200 fights. Most investigations focused on male cohorts; women appeared only in mixed-sex karate and fencing studies, making up roughly one quarter of the sample. Age ranged from 15 years in the youngest amateur boxers to 49 years in older judoka and retired professionals, with mean values clustering in the early to mid-twenties for active competitors. Training history varied widely. Where reported, amateurs averaged 3–7 years of experience, while elite athletes typically had more than a decade of continuous training and annual international competition. All studies excluded participants with known neurological or psychiatric conditions and those with recent concussion; several also required abstinence from psychoactive drugs, caffeine, or alcohol within 24–72 h of testing [48,49,59]. Control groups varied: eight studies matched non-athletes by age and education [45,46,47,49,51,52,53,54], three used athletes from non-contact sports such as soccer or track and field [42,45,47], and six relied on laboratory normative databases compiled from healthy volunteers [39,40,43,44,48,50]. This diverse yet well-screened pool of participants provides a broad, representative foundation for analyzing how repeated head impacts and high-level skill acquisition shape electrophysiological brain markers in combat sports.

### 3.2. EEG Recording Parameters

The electrophysiological methods used across the 23 included studies evolved markedly over the past four decades, moving from low-density, visually scored traces to high-density caps combined with graph-theoretical analytics.

#### 3.2.1. Montage and Channel Count

The earliest boxing and judo studies employed 14- to 21-lead clinical montages using Ag/AgCl cup electrodes placed according to the international 10–20 system [39,42]. Two boxing studies did not specify derivations but referred to “standard clinical” arrangements typical of late-1970s CT-EEG practice [40,41]. Beginning with 21-lead resting-state recordings in amateurs [42], subsequent research shifted to full 19-channel 10–20 QEEG for subconcussive effects [43,50] and 9-electrode frontal-to-parietal arrays for rapid ringside assessments in kickboxers [48,49,59,60,61]. From 2010 onward, studies of balance, posture, and network connectivity adopted high-resolution caps—either 56 active channels spaced in the extended 10–10 system [51,54,55,56,57,58] or 64 channels covering the entire scalp [44]. One attention study intentionally reduced spatial resolution to a single midline Cz channel to isolate theta/beta and theta/SMR ratios [45].

#### 3.2.2. Acquisition Conditions

Resting-state recordings (eyes-closed, eyes-open, or both) were used in 14 studies [39,40,42,43,44,45,46,47,48,49,50,52,54]. Seven investigations embedded EEG within functional paradigms: karate video judgment [51], mental arithmetic [53], monopodalic stance [55], quiet-stance visual withdrawal [56], cortico-muscular coupling during stance [57], self-paced wrist extension [58], and pre- versus post-bout monitoring [60,61]. Recording durations ranged from 1 min blocks in the theta/SMR study [45] to 30 min of continuous eyes-closed/intermittent-photic EEG in the Swedish boxing safety trial [42]. Modern graph-theoretical studies adopted 5 to 7 min eyes-closed protocols, yielding 80–120 artifact-free 2 s epochs per participant after data cleaning [43,44].

#### 3.2.3. Sampling Rate, Filtering, and Artifact Control

All contemporary datasets were digitized at ≥250 Hz, with high-density recordings sampled at 512 Hz [51,54,55,56,57]. High-pass filters were set between 0.1 and 1 Hz and low-pass filters between 30 and 45 Hz, with 50/60 Hz notch filtering when required. Investigations using more than 19 electrodes incorporated electrooculographic and electromyographic channels, along with independent component analysis (ICA), to excise ocular and muscle artifacts [43,44,51,54,55,56,57,58]. Earlier studies relied solely on visual rejection of contaminated EEG segments [39,40,41,42].

#### 3.2.4. Spectral and Connectivity Analysis

Clinical studies before 1990 relied on visual scoring of background rhythms and epileptiform discharges [39,40,41,42]. Later studies embraced fast Fourier transform (FFT)-based spectral analysis across standard frequency bands—delta (0.5–4 Hz), theta (4–8 Hz), alpha (8–13 Hz), sensorimotor rhythm (12–15 Hz), beta-1 (15–20 Hz), and beta-2 (20–35 Hz) [43,46,48,49]. Two investigations analyzed the 1–45 Hz spectrum using one-second Hamming windows with 50% overlap, extracting non-linear indices such as power-law exponent, detrended fluctuation analysis, long-range temporal correlations, and multiscale entropy [43,44]. The 64-channel boxing study generated phase-locking value matrices, applied network-based statistics to locate frequency-specific hyperconnected subnetworks, and quantified global and local efficiency, clustering coefficient, and small-worldness [44]. Balance-related projects derived event-related desynchronization (ERD) and task-related power decrease (TRPD) in individual alpha frequency-adjusted sub-bands (IAF−2→IAF; IAF→IAF+2) and localized sources using standardized low-resolution electromagnetic tomography (sLORETA) [51,54,55,56,58]. For cortico-muscular coupling, coherence and directed transfer function analyses were applied between 56-channel EEG and tri-axial EMG in the alpha and beta ranges [57].

#### 3.2.5. Reference Schemes and Spatial Transforms

Linked-earlobe or average reference montages were most commonly used (15 studies). High-density node-mapping studies re-referenced the data using a surface Laplacian or current-source density approach to minimize volume-conduction artifacts and enhance topographic precision prior to graph-metric extraction [44,55,56,57].

### 3.3. Time of EEG Recording in the Included Studies

Across the 23 papers, EEG was always obtained outside the moment of active striking. Four distinct recording windows emerge.

#### 3.3.1. Chronic “Off-Ring” Baselines (≥24 h After the Last Bout)

Long-interval resting EEG predominated: professional and amateur boxers or judoka were examined ≥1 month post-fight [39]; retired boxers years after retirement [40]; active amateurs during routine club visits, not around competition [41]; Swedish ex-amateurs decades after their careers [42]; and all modern resting-state or network studies in boxers, wrestlers, and kickboxers [43,44,45,46,47,48]. These sessions were scheduled in quiet laboratories, often with eyes closed, to characterize long-term adaptations or damage free from acute fatigue.

#### 3.3.2. Laboratory Tasks and Short Controlled Maneuvers

A second group recorded EEG while athletes performed discrete cognitive, motor, or balance tasks—video judging, arithmetic, stance, and wrist extension—or immediately before and after an 8 s juji-jime choke [50,51,52,53,54,55,56,57,58]. These protocols still occurred days to weeks from sparring, in shielded rooms, and served to probe neural efficiency or transient vascular effects without the artifacts of full contact.

#### 3.3.3. Immediate Pre-Contest Readiness

Only one dataset captured the psychophysiological state minutes before competition: K-1 kickboxers underwent 10 min eyes-open/eyes-closed QEEG in the locker-room just before entering the ring [59].

#### 3.3.4. Acute Post-Combat Recovery

Two studies measured brain activity within three minutes of the final bell. Professional kickboxers were wired during warm-up and re-tested ringside after real fights or after a punchbag simulation [60]; the same design was repeated with concurrent hormone sampling to link cortisol surges to frontal SMR and β_1_ power increases [61].

No study succeeded in recording during an exchange of blows: vigorous head movement, sweat, and the risk of electrode damage precluded in-fight acquisition. Instead, researchers have focused on (i) long-term baselines, (ii) controlled laboratory challenges, and (iii) the brief windows immediately before or after live competition to infer both chronic and acute brain responses in combat sport athletes.

### 3.4. Electrophysiological Outcomes

#### 3.4.1. Multimodal Imaging Cohorts (Boxing vs. Judo)

Early multimodal investigations that combined EEG with cerebral perfusion or structural imaging laid the groundwork for linking repetitive head impacts to objective brain dysfunction in combat sport athletes. In the largest of these studies, 20 active professional boxers and 10 high-level judoka underwent xenon-133 single-photon emission CT alongside 19-lead EEG recording. A quarter of the boxers showed global cerebral blood-flow values more than two standard deviations below age norms (53.5 ± 7.4 vs. 58.6 ± 5.5 mL·100 g^−1^·min^−1^; *p* < 0.01), and 35% exhibited fronto-central hypoperfusion that sometimes reached the occipital pole. Yet overt EEG slowing was evident in only two boxers; all perfusion and EEG abnormalities were confined to the boxing group, whereas judoka—who experience infrequent concussion but repeated chokehold exposure—displayed entirely normal perfusion maps and EEG traces [39].

A North American series of 40 retired boxers corroborated these perfusion findings. Computed tomography scans revealed ventricular dilatation that increased linearly with total bout count (χ^2^ trend = 10.7, *p* < 0.01), and diffuse or focal EEG abnormalities were more common among fighters with longer careers. Notably, neither CT nor EEG scores correlated with the number of knockouts sustained or with bedside neurological signs, highlighting the limited sensitivity of symptom-based screening [40]. In 20 active Scottish amateurs (mean 9 ± 5 fights), conventional EEG detected low-frequency (≤7 Hz) or focal delta–theta activity in 40% of participants, but these changes correlated inversely with age (r = −0.47, *p* < 0.05) rather than with fight exposure and were seldom accompanied by imaging pathology (19/20 CT scans normal), suggesting that mild slowing can precede structural change [41].

Finally, a Swedish safety audit of 47 former amateurs competing under modern rules compared high-match (>50 bouts) and low-match (≤10 bouts) boxers with soccer and track-and-field controls. Mild or moderate EEG deviations—chiefly diffuse alpha instability or intermittent slow bursts—appeared in 32% of high-match and 36% of low-match boxers, versus 20% of soccer and 12% of athletic controls. Brain electrical activity mapping topographies and brainstem auditory-evoked potentials were identical across all groups, and no athlete exhibited severe abnormalities [42].

Taken together, these studies show that global or regional hypoperfusion and ventricular enlargement are detectable only in boxers, and that the prevalence of EEG slowing scales with total bout count rather than with knockout history. Judoka, despite repeated transient hypoxia from chokeholds, show no chronic perfusion or EEG deficits, while amateurs competing under contemporary safety regulations display only subtle, non-progressive EEG variants, suggesting that modern rules and shorter exposure windows may mitigate long-term electrophysiological damage.

#### 3.4.2. Spectral and Network Changes in Boxing

High-resolution spectral analyses reveal that even cognitively intact boxers accumulate subtle resting-state abnormalities once their career exposure exceeds roughly two dozen contests. In a rigorously screened cohort of 21 amateurs, those with ≥25 bouts showed a 14–18% reduction in normalized theta (4–7 Hz) and alpha (8–13 Hz) power at 15 of 19 scalp electrodes (Wilcoxon *p* < 0.01), a local flattening of the scale-free power-law exponent at F3 (ΔPLE = −0.12, *p* = 0.004), and a shortening of long-range temporal correlations in the alpha envelope (DFA-α 0.72→0.64, *p* = 0.03) [43]. Collectively, these linear and non-linear shifts reproduce the electrophysiological signature seen in prodromal Alzheimer’s disease, suggesting that microstructural degeneration may be underway well before measurable cognitive decline in contact sport athletes.

Graph-theoretical EEG applied to 24 young boxers confirmed early network disintegration. Across theta, beta, and gamma bands, phase-locking value rose by 12–18% (false-discovery rate *q* < 0.05), yet global efficiency fell by 9% and characteristic path length increased by 14%; simultaneously, hub nodes migrated from the left temporal lobe to right parieto-occipital regions, and the small-worldness coefficient dropped below the healthy range (σ = 0.98 ± 0.05 vs. 1.12 ± 0.07, *p* < 0.001) [44]. This pattern—heightened local synchrony coupled with impaired long-range integration—implies compensatory over-coupling of nearby cortical generators at the expense of efficient distributed processing, reinforcing the notion that repetitive subconcussive blows alter functional architecture long before overt neuropsychological deficits emerge.

#### 3.4.3. Sport-Specific Resting Profiles

Single-channel and multichannel studies show that each combat discipline leaves a distinct resting-state EEG fingerprint. In boxing, Cz recordings from successful amateurs reveal a clear attentional bias: theta amplitude falls to 2.6 ± 0.9 µV while SMR rises to 4.3 ± 1.2 µV compared with 3.4 ± 1.1 µV and 3.5 ± 1.1 µV in less accomplished peers, producing ~15% lower theta/beta and theta/SMR ratios—metrics generally interpreted as more efficient sustained attention [45]. A separate seven-lead comparison, though under-powered, echoed this pattern with a uniform downward shift in alpha power that peaked at −23% at O2 and −19% at Cz, hinting that habitual sparring may desynchronize posterior cortices even in the absence of measurable deficits [46].

Elite wrestlers exhibit an opposite but equally consistent modulation of occipital rhythms. Their absolute alpha power is 17–21% lower than that of non-athletes under both eyes-open and eyes-closed conditions, yet the coefficient of reactivity (CR = alpha_closed/alpha_open) climbs to 1.20 ± 0.17 vs. 1.00 ± 0.14 in controls (*p* < 0.05). This heightened desynchronization to visual input—and rapid rebound once the stimulus is removed—suggests a training-induced sharpening of visuomotor gating, enabling wrestlers to switch efficiently between externally orienting and internally predictive modes [47].

Kickboxers, evaluated with nine-lead quantitative EEG, display a third, hyper-vigilant pattern. At rest with eyes closed, delta at Fz breaches the 20 µV normative ceiling in 44% of athletes, theta approaches 15 µV in 39%, and SMR, β-1, and β-2 exceed 6 µV in more than 80%—values typically linked to sustained arousal. Opening the eyes fails to dampen this activation: frontal β-1/β-2 remain elevated (Mann–Whitney *p* < 0.01), and marked F3–F4 asymmetries (>20% for alpha) point to right-hemisphere dominance, a hallmark of threat monitoring and rapid action readiness [48,49].

#### 3.4.4. Task-Evoked and Perturbation Studies

##### Neural Efficiency Tests in Karate

Eight tightly controlled experiments chart how elite karate practitioners attain technical mastery with measurably less cortical expenditure—a core prediction of the neural efficiency hypothesis. In the first series, twelve national-level judges, twelve advanced amateurs, and twelve non-athletes watched ten unfamiliar kata sequences and rated their quality. Although the experts reproduced the coach’s scores most faithfully (median Spearman *r* = 0.40 vs. 0.19 vs. 0.04), they generated ≈ 30% smaller event-related desynchronization (ERD) in the high-alpha band (10–12 Hz) across dorsal visuospatial areas (BA 7/5/6d) and fronto-parietal “mirror” circuits (BA 6v/40/44–45) throughout the 1–7 s evaluative window [51]. The same athletes, recorded at rest, displayed 1.7–1.9-fold higher parieto-occipital alpha-1 (8–10 Hz) power together with a central–parietal delta surplus—an oscillatory configuration previously linked to top-down gating and effortless attentional readiness [52].

When cognitive load was manipulated directly, fifteen black belts and fifteen controls subtracted serial threes aloud for three minutes. Experts achieved identical accuracy but required 20–25% less alpha and beta ERD across frontal, centro-temporal, and parieto-occipital sites (group × task interaction *p* < 0.003) [53]. A simple eyes-closed→eyes-open transition evoked the same pattern: alpha total-root-power depression (TRPD) was 15–30% smaller at F3/Fz/F4, Cz/C4, and O2 in karateka, confirming that even rudimentary sensory gating is metabolically cheaper for them [54].

Motor and postural challenges yielded parallel results. During single-leg stance, the experts’ high-alpha ERD fell by just 10–18% at C3/C4/Pz/P4 (controls: 25–32%, *p* < 0.008) [55]; in self-paced wrist extension, desynchronization in the primary motor hand area (BA 4) reached −24% versus −38% in novices (*p* = 0.002) [58]. Conversely, when balance relied heavily on vision—achieved by closing one eye and tilting the platform—karateka exhibited greater high-alpha ERD over the right parietal cortex (CP6/C6), and the magnitude of this desynchronization correlated with sway area reduction (*r* = 0.61, *p* = 0.008), underscoring context-specific up-regulation when additional resources genuinely improve performance [56]. Finally, cortico-muscular coherence analyses during quiet stance revealed that non-athletes display higher alpha-band EEG–EMG coupling with the gastrocnemius, whereas athletes maintain lower coherence yet preserve strong cortex→muscle directed transfer entropy. This pattern indicates more selective, fine-tuned descending control consistent with economical neural drive [57].

#### 3.4.5. Transient Grappling Maneuvers

The only study to probe the moment-to-moment neural repercussions of a chokehold used a standardized juji-jime application administered to experienced judoka while nine-lead QEEG was recorded continuously before, during, and for 90 s after release. The carotid–jugular compression lasted 8 ± 1.8 s—short enough to avoid syncope yet long enough to provoke a brief cerebrovascular challenge. Within the first 3–20 s of recovery (Phase BI) global delta–theta power surged by 28% (Wilcoxon *p* < 0.01), signaling transient cortical deafferentation or mild hypoxia. Simultaneously, occipital alpha (8–13 Hz) fell by 16%, reflecting a loss of posterior idling that typically accompanies heightened subcortical drive to restore cerebral perfusion. Crucially, both metrics decayed mono-exponentially and returned to baseline within ~70 s, and no participant reported dizziness, exhibited epileptiform discharges, or failed a brief post-test coordination screen [50].

#### 3.4.6. Acute Competition Responses

Electrophysiological monitoring taken immediately before and after real contests demonstrates that receiving head strikes triggers a rapid, dose-dependent shift toward slower and faster extremes of the EEG spectrum—an effect not reproduced by non-contact simulations. In a field study of 50 professional K-1 kickboxers, nine-lead QEEG was recorded at three time points: (i) 10 min pre-bout, (ii) ≤5 min post-bout, and (iii) the same schedule during a controlled punchbag session on a separate day. Pre-fight baselines already differed from population norms: frontal theta (4–7 Hz) at Fz averaged 12.8 ± 3.1 µV—at the upper 95% reference limit—while beta-2 (20–30 Hz) at F4 peaked at 10.2 ± 2.6 µV, exceeding the normative mean by >2 SD. With eyes closed, these values rose a further 9–12%, consistent with task-independent internal visualization and sympathetic arousal [59].

Actual competition amplified these abnormalities. Immediately post-bout, delta (1–4 Hz) and beta-2 amplitudes climbed by Δ + 6.1 µV and Δ + 4.8 µV, respectively, across frontal and occipital leads, altering 74–78% of all QEEG parameters relative to the athletes’ own warm-up baselines. The magnitude of change correlated positively with the total number of head strikes received (Spearman ρ = 0.53–0.61, *p* < 0.01), indicating that both very slow and very fast oscillations scale with acute cranial load rather than general exertion [60].

Endocrine measurements collected in parallel linked the electrophysiological response to stress axis activation. Serum cortisol increased from 313 ± 82 nmol·L^−1^ pre-bout to 570 ± 105 nmol·L^−1^ post-bout (*p* < 0.001), whereas testosterone fell modestly (17.9 ± 4.3→15.6 ± 4.0 nmol·L^−1^, *p* = 0.04). The resulting drop in the testosterone-to-cortisol ratio (T/C) predicted individual gains in frontal sensorimotor rhythm (SMR, 12–15 Hz) and beta-1 power (15–20 Hz): Kendall’s τ = 0.32–0.36, *p* < 0.05. These associations implicate glucocorticoid-driven vigilance and inhibitory control mechanisms in shaping the immediate neural aftermath of head impact exposure [61].

By contrast, a matched punchbag simulation—identical in duration, round structure, and perceived effort but devoid of cranial impacts—elicited only marginal endocrine shifts (cortisol + 11%, testosterone − 3%) and left ≥ 85% of QEEG variables unchanged. Together, these findings show that the unique combination of mechanical head trauma and acute psychophysiological stress in live competition produces a transient but pronounced bifurcation of EEG power toward delta and high-beta bands, tightly coupled to both strike count and hormonal milieu—an electrophysiological signature absent when athletes strike but are not struck.

Taken together, the evidence delineates a continuum—from rare but clear low-frequency slowing and hypoperfusion in veteran fighters, through to quantitative reductions in alpha–theta power and network efficiency that manifest before cognitive symptoms, to a distinctive efficiency profile in elite athletes who achieve equal or superior task performance with less cortical activation. Superimposed on these chronic adaptations, acute fights provoke transient delta- and beta-range hyper-activity tightly linked to stress hormone release and blow count, whereas brief vascular occlusion (e.g., judo chokes) results in only short-lived slow-wave bursts without sustained dysfunction. Despite remaining methodological heterogeneity, the emerging pattern confirms EEG’s utility in capturing both the detrimental impact of repetitive head trauma and the adaptive neuroplasticity associated with elite combat sport expertise.

**Table 1:** Studies included in review.

Key EEG Findings	Analysis	EEG Set-Up	Control/Comparison	N	Sport/Population	Study
Slowing in 2 pros and 1 amateur; none in judoka; matched regional hypoperfusion	Visual scoring	14 ch, eyes-closed rest	Age- and sex-matched healthy	44 boxers + 10 judoka	Boxers (pro and am) + judoka	[39]
Abnormalities ↑ with number of bouts; no link to symptoms	Visual (+CT)	Clinical EEG (details NR)	None	40 (24 EEG)	Former boxers	[40]
Abnormal EEG in 8 (40%); associated with younger age; 4 overlap with neuro signs	Visual	Clinical EEG	None	20	Active amateur boxers	[41]
Slight/moderate deviations in 32–36% of boxers; no severe or exposure correlation	Visual + BEAM	21 ch, rest + photic	Soccer and track–field	47 boxers + 25 soccer + 25 track	Former amateur boxers (HM/LM)	[42]
Advanced: ↓theta/alpha power, ↓ PLE and LRTC; AD-like entropy shift	FFT + PLE, DFA, MSE	19 ch, 7 min EO/EC	Within-sport groups	21	Amateur boxers (beginner vs. advanced)	[43]
Boxers: hyperconnectivity but ↓ global/local efficiency and small-worldness	PLV and graph theory	64 ch, 5 min EC	Non-athletes	24 boxers/25 ctr	Active boxers vs. controls	[44]
Boxers show lower ratios→better attentional indices	Theta/β and Theta/SMR ratios	Single Cz, 1 min each cond.	Phys-ed students	36/52	Amateur boxers vs. students	[45]
Non-sig trend to lower alpha power in boxers	FFT (alpha)	13 ch, 3 min EO/EC	Non-athletes	7/9	Amateur boxers vs. sedentary	[46]
↓ Alpha power and ↑ reactivity coefficient in wrestlers	Band power, reactivity	C3 and C4, EC/EO/EC	Non-athletes	30/30	Elite wrestlers vs. controls	[47]
Elevated alpha, SMR, β; frontal asymmetry and hyper-arousal profile	QEEG (FFT)	9 ch, EC	None	18	Elite K-1 kickboxers	[48]
Kickboxers: ↑ δ, θ, α, β across scalp; SMR ↑ Cz/P3	QEEG	9 ch, EO	Non-athletes	18/18	Elite K-1 vs. controls	[49]
Post-choke: ↑ δ/θ, ↓ α (occipital); resolves < 70 s	Spectral	19 ch, EC	Pre- vs. post	6	Judoka (juji-jime choke)	[50]
Elite: lower ERD in dorsal and MNS→greater neural efficiency; aligns with rating accuracy	Alpha ERD, sLORETA	56 ch	3-group	16/15/17	Karate (elite/amateur/NA) judgment task	[51]
Elite: ↑ parietal/occipital α1 and δ/θ; trait marker of expertise	LORETA sources	56 ch, 3 min EC	Multiple	21/23/30	Karate (elite/amateur/NA) rest (+ gymnast ctrl)	[52]
Athletes keep higher α/β and lower ERD under load	α/β ERD/ERS	16 ch, EC rest→math	Matched controls	10/10	Elite karate vs. NA (mental arithmetic)	[53]
Smaller TRPD (less α desync) in athletes→reduced cortical reactivity	Alpha TRPD	56 ch	Non-athletes	18/28	Elite karate vs. NA (EO vs. EC)	[54]
Athletes show lower desync during demanding stance	Alpha TRPD	56 ch, bipodalic vs. monopodalic	Multi-group	10 KAR/10 FEN/12 NA	Karate and fencing elites vs. NA (posture)	[55]
Only elites: strong parietal α ERD correlates with visual balance gain	Alpha ERD + sway	56 ch	Multi-group	19 KAR/18 FEN/10 NA	Karate and fencing elites vs. NA (stance EO/EC)	[56]
Elites: lower α cortex-muscle coherence; stable cortex→muscle drive	EEG-EMG coherence and DTF	56 ch + EMG	Multi-group	19 EL/14 AM/18 FEN/9 NA	Karate (elite/am) + fencing vs. NA	[57]
Reduced α ERD in motor areas during movement	Alpha ERD, sLORETA	56 ch	Non-athletes	10/12	Elite karate vs. NA (wrist movement)	[58]
High β2 and θ; amplitudes higher with eyes closed→internal rehearsal/arousal	Theta and β2 amplitude	9 ch	EO vs. EC within-subj	15	K-1 kickboxers pre-fight	[59]
Post-fight: ↑ δ and β2; magnitude correlates with head blows	QEEG bands	9 ch, pre and ≤3 min post	Punchbag simulation	50 fight/50 bag	K-1: fight vs. bag	[60]
Fight: ↑ SMR and β1 (F3/P3) with cortisol surge; T/C ratio drops	QEEG + T/C ratio	9 ch, pre and post	Punchbag simulation	50 fight/50 bag	K-1: fight vs. bag (+hormones)	[61]

Abbreviations: AM—amateur; BEAM—brain electrical activity mapping; β/β1/β2—beta-band EEG activity (13–20 Hz = β1; 20–35 Hz = β2); CT—computed tomography; CTR/Ctr—control participants; δ—delta-band EEG activity (0.5–4 Hz); DFA—detrended fluctuation analysis; DTF—directed transfer function; EEG—electroencephalography; EL—elite athletes; EMG—electromyography; ERD—event-related desynchronization; FFT—fast Fourier transform; HM/LM—high match/low match; LRTC—long-range temporal correlation; LORETA/sLORETA—(standardized) low-resolution electromagnetic tomography; MNS—mirror neuron system; MSE—multiscale entropy; NA—non-athletes; PLV—phase-locking value; PLE—power-law exponent; QEEG—quantitative EEG; SMR—sensorimotor rhythm (12–15 Hz); σ, λ, γ (small-world)—graph-theory small-world metrics; θ—theta-band EEG activity (4–8 Hz); TRPD—task-related power decrease.

## 4. Discussion

The present review synthesizes four decades of electrophysiological research in combat sports, encompassing classic visual EEG, quantitative spectral analysis, non-linear metrics, and modern graph-theory approaches. Collectively, the 23 studies reveal a complex—and sometimes apparently contradictory—landscape in which signs of cumulative brain injury and markers of adaptive neural optimization coexist.

### 4.1. Neural Efficiency: A Protective or Confounding Factor?

Elite karate and fencing athletes consistently exhibit smaller task-related alpha desynchronization (ERD) during cognitive, sensory, and motor challenges [51,53,54,55,56,57,58]. This pattern matches the neural efficiency hypothesis (NEH). The NEH originated from Richard Haier’s positron emission tomography work in the late 1980s, which showed that people who performed better on Raven’s advanced progressive matrices used less glucose in the very cortical regions engaged by the task—leading to the idea that higher ability correlates with reduced metabolic expenditure [62]. Since then, evidence from PET, fMRI, multichannel EEG, and MEG has expanded that insight [63]. Across modalities, the hallmark of efficiency is always relative: when individuals with higher intellectual or motor proficiency—or those further along in learning—confront a low-to-moderately demanding task, they display either lower hemodynamic responses, higher task-related alpha power, or weaker event-related desynchronization in precisely those pre-frontal and inferior parietal areas that govern reasoning, working memory, or fine sensorimotor control [63]. Longitudinal learning studies complement the cross-sectional portrait, showing that activation is initially diffuse and costly but becomes progressively focal and economical as strategic routines crystallize [64].

Sport neuroscience has broadened the scope of this theory. Systematic surveys covering 28 studies in 18 disciplines—from rifle and pistol shooting to archery and fencing—consistently find that experts, compared to novices, exhibit consistently higher alpha power and reduced sensorimotor cortical activity in the final seconds before the trigger pull or arrow release, a pattern that correlates with better performance accuracy [65]. Training-season studies indicate that the same shooter’s brain shifts from broad beta engagement to a quieter left temporal and parietal focus as accuracy stabilizes, confirming that efficiency can be acquired through practice [66]. Meta-analyses of self-paced sports add further nuance: optimal performance is marked not only by widespread alpha enhancement but also by modest frontal theta suppression, implying a temporary relaxation of executive monitoring—a phenomenon Dietrich described as transient hypofrontality [64].

However, efficiency is not always beneficial. When task complexity approaches the person’s limit, the relationship may reverse: high-ability participants may recruit more neural resources, an observation known as the “efficiency paradox” [65]. Variables such as sex, brain region, and learning stage also influence efficiency. The effect is most prominent in anterior association cortices, whereas sensory regions may require equal or even heightened engagement in experts due to their refined perceptual demands [63]. These complexities led to the broader concept of neural proficiency—the idea that experts can down-regulate irrelevant processing to conserve energy but can rapidly engage executive control, integrating neural efficiency, transient hypofrontality, and strategic resource deployment into a flexible, adaptive system [64].

Multiple neurobiological mechanisms appear to support this efficiency. First, a structural capacity mechanism: highly able or highly trained individuals often possess greater regional gray- or white-matter volume, especially along the parieto-frontal integration (P-FIT) circuit that supports complex problem solving [63]. While greater volume might imply more energy use, it may reduce per-synapse energy demands and create reserve capacity for high-load tasks [63]. Denser myelination would also speed conduction, allowing shorter bursts of activity to accomplish the same computation. Second, a metabolic selection mechanism refines this potential in real time. Across PET, fMRI, and near-infrared studies, high-performing individuals show focal decreases in BOLD or blood-flow signals in pre-frontal areas during low-to-moderate challenges, yet they can up-shift those signals when difficulty soars, producing the “efficiency paradox”. Training amplifies this effect; after weeks of Tetris practice, whole-brain glucose utilization falls markedly, especially in the most improved learners [63]. Third, efficiency is enforced by an inhibitory control mechanism. Doppelmayr’s “inhibition hypothesis” holds that able brains excel at suppressing task-irrelevant circuits, a skill reflected in stronger event-related synchronization (ERS) of alpha rhythms over those regions [63]. Alpha ERS is widely interpreted as a sign of functional inhibition [67]; accordingly, elite marksmen show surges of alpha power in the occipital cortex just before the trigger pull, muting distracting visual input and sharpening the sensorimotor loop [66].

Fourth, there is a network-optimization mechanism. EEG coherence analyses reveal that experts reduce long-range chatter among associative hubs; for example, elite shooters display weaker coupling between left temporal (T3) and frontal midline (Fz) sites during the 4 s aiming phase, indicating a leaner, more segregated network architecture [66]. Meta-analyses of self-paced sports confirm this, showing global increases in alpha and decreases in theta during optimal attempts; the frontal lobe in particular becomes both “quieter” (higher alpha) and “less busy” (lower theta)—a transient hypofrontality that frees posterior circuits to run the skill automatically [64]. Fifth, an oscillatory gating mechanism fine-tunes resource allocation within milliseconds. Event-related desynchronization (ERD) in the alpha band scales with processing load; brighter people display smaller ERD (i.e., retain more alpha) when the load is modest, again signaling a thrifty deployment of cortical columns [63]. Theta power, in contrast, rises frontally when executive control is truly required; the meta-analysis shows that only under the heaviest demands do experts allow frontal theta to climb, preserving precision without habitual over-recruitment [64]. Finally, an experience-dependent redistribution mechanism shifts the burden from general executive to specialized posterior regions as the performance of a skill becomes automatic. After extensive chess or taxi-navigation practice, frontal activity diminishes while parietal, temporal, or cerebellar foci intensify, reflecting the transfer of control to domain-specific representations that run with minimal supervisory cost.

Taken together, these mechanisms—structural capacity, metabolic selection, inhibitory control, network optimization, oscillatory gating, and experience-driven redistribution—offer a coherent neurobiological explanation of how expertise reduces cortical effort. They also define the boundary conditions of the NEH: efficiency is most pronounced in the frontal cortex, during low-to-moderate tasks, and after sufficient learning, but it may reverse in novel or complex contexts.

Returning to combat sports, two observations complicate an unreservedly positive reading. First, higher resting alpha is not always beneficial—it also appears in post-traumatic stress and chronic pain. Second, elite fighters display exaggerated delta–theta amplitudes at rest [48,49,52], a feature more typical of mild traumatic encephalopathy [68,69,70] than of healthy optimization. Whether neural efficiency offsets, coexists with, or masks early neuropathology remains unresolved.

Modern intracranial and MEG research indicates that alpha oscillations enact pulsed inhibition: 8–13 Hz cycles rhythmically silence pyramidal firing, thereby gating information flow and reducing metabolic demand [71]. Crucially, computational models propose that alpha power is indirectly governed by the load of goal-relevant information—the greater the precision of internal predictions, the stronger the suppression of distracting input [72]. In elite performers, extensive training compresses sensorimotor predictions into tightly tuned generative models, allowing larger swathes of cortex to maintain an inhibited, high-alpha state until truly task-critical information arrives. Reduced ERD may therefore reflect selective disinhibition of well-practiced modules rather than global under-engagement. Elevated resting alpha is also associated with greater cortical GABA concentration [73], suggesting a neuroprotective effect. By minimizing synaptic firing and oxidative load, neural efficiency may buffer the brain against the metabolic stress of high-volume practice. However, blunted alpha reactivity may also emerge after thalamo-cortical disconnection in chronic mTBI—indistinguishable at the scalp level from genuine efficiency. Without concomitant behavioral or structural data, apparent “economy” could mask latent pathology, making neural efficiency a potential confounding factor when interpreting EEG screening in contact sport athletes.

### 4.2. Evidence for Trauma-Related Dysfunction

Early research in professional boxing demonstrated that repeated head impacts can culminate in fronto-central hypoperfusion and low-frequency EEG slowing, even in athletes with normal neurological exams [39,40,41]. Later QEEG studies refined these findings, showing that reduced alpha- and theta-band power [43,46,47] and loss of small-world organization in theta, beta, and gamma networks [44] are among the most sensitive resting-state correlates of repetitive head impact. Importantly, these alterations emerge before cognitive or structural abnormalities are detectable on standard tests or CT. Acute competition amplifies chronic changes—surges in delta and high-beta activity with the number of head strikes [60] and a rise in cortisol [61]—indicating that repeated “spikes” in physiological stress may accelerate long-term network degeneration.

Although this review focuses on combat sport athletes, the much broader neuro-trauma literature confirms that EEG is highly sensitive to both acute and chronic consequences of mTBI [70]. Findings from retired contact sport athletes, blast-exposed soldiers, and civilians with TBI converge on four recurring electrophysiological signatures.

A recent systematic review of resting-state EEG after sport-related concussion reported that every eligible chronic-phase study demonstrated reduced alpha (8–13 Hz) and increased delta (0–3 Hz) power in symptomatic athletes, even in athletes cleared for return to play [74]. These spectral changes align with the neurometabolic cascade of concussion and are detectable weeks to months after injury, underscoring their usefulness as a lagging biomarker of incomplete recovery.

In 87 retired National Football League players, a machine learning algorithm built from 50 qEEG features correctly distinguished ex-professionals from unexposed peers with 80% specificity. The discriminating pattern combined theta–delta slowing with disrupted alpha-band connectivity, and the magnitude of abnormality scaled with cumulative exposure (age of first tackle in football < 12 years vs. ≥12 years) [75]. These results indicate that electrophysiological sequelae of repetitive head impacts (RHIs) can be detected decades after the last competitive season.

Phase-synchrony analysis in soldiers several months after blast concussion revealed diminished inter-hemispheric frontal synchrony that correlated with fractional anisotropy loss in the anterior corpus callosum, even in the absence of neuropsychological deficits [76]. Similarly, active-duty service members assessed within three years of mTBI showed selective reductions in low-gamma (25–40 Hz) synchrony, and the degree of gamma desynchronization tracked white-matter damage in the inferior cerebellar peduncle [77]. These studies provide direct evidence that qEEG abnormalities can index microstructural axonal injury rather than secondary psychiatric factors.

Graph-theoretical examination of EEG recorded during unpredictable balance perturbations demonstrated that individuals with chronic mTBI fail to down-regulate network segregation in the alpha band and had lower global efficiency. Greater theta-band segregation was tied to worse postural stability [78]. Such task-dependent network inflexibility mirrors the reduced small-worldness observed at rest in head impact cohorts and offers a mechanistic explanation for latent motor-control deficits.

### 4.3. Pathophysiological Integration

Taken together, the literature supports a two-process model. Repetitive head trauma initially disrupts large-scale communication efficiency—manifest as reduced alpha/theta power, hyper-synchrony, and longer path length—while cognition remains intact. In parallel, athletes who reach elite levels refine cortical circuits to improve signal-to-noise ratios and require less desynchronization for a given task. The two trajectories are not mutually exclusive: efficiency may partially compensate for trauma-induced inefficiency, delaying clinical expression. Acute bouts or chokeholds then produce transient vascular–metabolic stress (delta–theta bursts, alpha suppression) which, over time, may exceed the brain’s compensatory capacity.

A coherent mechanistic picture is emerging in which the diverse electrophysiological signatures seen after repetitive head impact—slow-wave excess, alpha attenuation, beta/gamma hyperconnectivity, and graph-level inefficiency—correspond to specific biological lesions that unfold over time.

Mechanical strain on long axons is the dominant primary injury in concussion. Plasma neurofilament light (NfL), a quantitative marker of large-caliber axonal damage, increases in a dose-dependent fashion after mild TBI and remains elevated for months [79]. Athletes and soldiers with the highest NfL titers show the greatest loss of alpha-band coherence and lower small-worldness, linking microscopic axonal disruption to macroscopic network fragmentation. Diffusion MRI corroborates the relationship: reduced fractional anisotropy in the optic radiation predicts a downward shift in individual alpha-peak frequency [80], while children and young adults with thicker visual white-matter pathways exhibit faster alpha rhythms and better perceptual accuracy. In this context, conduction integrity along posterior cortico-cortical tracts appears to set the pace for alpha oscillations.

A systematic review of 23 arterial spin labeling (ASL) studies shows that thalamic and fronto-parietal hypoperfusion are among the most consistent chronic findings following mTBI. Decreased cerebral blood flow correlates with symptom severity and can persist beyond structural MRI normalization [81]. Because delta activity is highly sensitive to reduced perfusion [82], regional CBF loss likely explains the recurrent delta/theta elevations at fronto-central sites in retired fighters and blast-exposed personnel. Experimental studies in animals confirm the link: impaired reactivity following controlled cortical impact predicts increases in cortical delta power within days of injury [83].

Chronic neuroinflammatory processes progressively erode inhibitory circuitry. In a rat model of mild blast TBI, selective depletion of parvalbumin- and somatostatin-expressing interneurons in layer-5 cortex was accompanied by broadband power surges and gamma-band hyperconnectivity on chronic EEG—despite minimal acute EEG abnormalities [80]. These findings provide a cellular substrate for the persistent high beta/gamma activity recorded in some athletes and clarify how hyperconnectivity may arise alongside global efficiency: surviving pyramidal networks compensate for local inhibitory loss through excessive long-range synchronization.

Downstream of axonal and vascular injury, tau aggregation exacerbates neuronal silencing. Flortaucipir-PET scans in 70 retired contact sport athletes demonstrate focal uptake in dorsolateral frontal and medial temporal cortices that correlates with cortical thinning and executive deficits [84]. Regions with the largest tau burden overlap those that generate alpha rhythms; post-mortem studies confirm loss of thalamocortical projections in the same territories, creating a plausible link between PET-detectable tauopathy and alpha slowing on scalp EEG.

Since each type of biological injury leaves a partly distinctive electrophysiological signature, multimodal coupling—for example, qEEG with NfL (axons), ASL (perfusion), and PET-tau (proteinopathy)—could help stage an athlete’s brain along the continuum from injury to degeneration. Such staging would be critical to distinguish between adaptive neural efficiency and early pathology, guide return-to-play criteria, and identify a therapeutic window before irreversible damage to inhibitory circuits or tau accumulation occurs.

### 4.4. Discipline-Specific Phenotypes

Boxers and kickboxers sustain hundreds of direct head impacts. Across six independent cohorts, delta/theta power was 15–30% higher than in controls at fronto-central sites, while alpha power was 10–20% lower [43,46,48,49]. Graph-theory analysis showed 9–12% falls in global efficiency and loss of small-worldness in theta, beta, and gamma bands [44]. These chronic alterations were exacerbated immediately after bouts: frontal/occipital delta increases of ≈6 µV and beta-2 increases of ≈5 µV were strongly correlated with the number of recorded head strikes (ρ = 0.53–0.61) [60]. Similar frequency elevations occurred when athletes simply close their eyes pre-fight, suggesting an interaction between cumulative trauma and state anxiety [59].

Elite wrestlers demonstrated 17–21% lower absolute alpha but an alpha-reactivity coefficient (alpha_closed/alpha_open) 20% higher than non-athletes [47]. This indicates stronger desynchronization to visual stimuli and faster resynchronization—a sign of enhanced sensorimotor gating rather than generalized slowing. In contrast, long-term judoka, who experience thousands of transient carotid compressions, showed no evidence of chronic hypoperfusion or EEG slowing [39]. Controlled juji-jime chokes caused only a brief (≈20 s) delta/theta surge, which fully resolved within 70 s [50]. Overall, grappling appears to induce acute vascular stress without the progressive network degradation typical of repetitive head punches.

Karate combines strikes, kicks, throws, and kata sequences. At rest, national-level karateka showed parieto-occipital alpha-1 amplitudes almost twice as high as those of amateurs, alongside mild diffuse delta elevation [52]. During tasks, they displayed smaller alpha ERD (and beta ERD) than both amateurs and non-athletes, while achieving equal or better performance on tests of action judgment, arithmetic, single-leg stance, and self-paced wrist extension [51,53,54,55,56,57,58]. This profile—high resting alpha and reduced task-ERD—is commonly interpreted as neural efficiency. However, the concurrent delta excess mirrors findings in striking sports, implying that karate athletes may simultaneously develop adaptive circuit optimization and accumulate low-grade brain injury.

### 4.5. State-Dependent Confounders

In the reviewed literature, the same athlete could display EEG readings indicative of severe brain dysfunction in one moment and near-normal patterns moments later. This volatility is not artifact—it reflects the physiological and psychological volatility inherent to combat sports. Two transient modulators recur so consistently that any combat sport EEG recorded without tracking them is, at best, ambiguous and, at worst, misleading.

Alpha amplitude drops by 30–50% when the eyes open, yet four studies using resting-eyes-open protocols compared their findings to eyes-closed norms, erroneously labeling the alpha reduction as “pathological.” Conversely, neural efficiency studies often credit elite athletes with reduced ERD, without noting that participants were mentally rehearsing kata—a condition that preserves alpha power even with eyes open. Unless visual condition, gaze fixation, and mental task instructions are standardized, alpha metrics remain non-comparable across studies.

Physical factors also introduce bias. Protective head-gear, braided hair, Vaseline on brows, and even post-fight hematomas can alter electrode contact impedance and produce low-frequency drifts that mimic delta waves. Modern dry-sensor EEG systems, while convenient ringside, tend to amplify movement noise in the 20–30 Hz band—the same band where beta activity linked to stress is measured. Fewer than half of the reviewed studies reported electrode impedance values. Until standardized EEG hardware and protocols are adopted for combat sports, findings of beta hyperactivity should be interpreted cautiously.

### 4.6. Relationship Between EEG Abnormalities and Clinical or Functional Indices

Across the 23 studies reviewed, links between electrophysiological findings and “real-world” outcomes fall into three broad clusters—(i) exposure metrics and structural markers, (ii) clinical–neurological or cognitive status, and (iii) acute performance or stress variables. Overall, correlations are sporadic and context-dependent: large changes in resting or task-evoked EEG can coexist with normal clinical examination, whereas subtle electrophysiological differences sometimes track behavioral precision or hormonal load.

#### 4.6.1. Exposure Load, Imaging, and EEG

Two boxing cohorts demonstrated a dose–response relation between cumulative exposure and brain structure/function. In retired professionals, ventricular dilatation on CT and the prevalence of diffuse EEG abnormalities both rose linearly with bout number [40]. Similarly, post-fight delta and β2 surges in K-1 athletes scaled with the count of head blows received in the same bout (ρ ≈ 0.55) [60]. By contrast, the largest multimodal series found neither global CBF nor EEG slowing to correlate with fight history [39], and Swedish amateurs competing under modern rules showed no association between any electrophysiological metric and bouts, knockouts, or career length [42]. Hence, exposure correlates with EEG only when impacts are frequent and severe enough to produce macro-structural change or when blows are quantified very precisely; otherwise, the relation is weak or absent.

#### 4.6.2. Clinical Examination, Neuropsychology, and EEG

Classic neurological signs correlate poorly with electrophysiology. Retired boxers displayed clear CT/EEG deterioration without parallel changes in bedside examination [40], and Scottish amateurs showed EEG slowing tied to age rather than fight number, despite a significant fight-related rise in abnormal reflexes [41]. Cognitive test batteries tell a similar story: nine of fifteen amateurs were neuropsychometrically impaired, yet their EEG patterns were heterogeneous and the study reported no direct EEG-cognition statistics [41]; in advanced boxers, Alzheimer-like linear and non-linear spectral changes appeared while MoCA and FAB scores remained normal [43]; and network inefficiency in young boxers likewise emerged in the face of intact MMSE and IQ [44]. Collectively, resting-state EEG seems to identify pre-clinical brain change earlier than conventional exams, but published work offers little evidence of tight, continuous coupling between electrophysiology and standard cognitive scales.

#### 4.6.3. Performance-Specific or Stress-Related Couplings

When neural activity is sampled while athletes are actually thinking, balancing, or bracing for impact, the electrophysiological signal aligns much more tightly with behavior and physiology than it does at rest.

During a one-minute vigilance test, successful Polish amateurs displayed lower theta amplitude (≈2.6 µV) and higher SMR (≈4.3 µV) at Cz than age-matched students. The resulting theta/β and theta/SMR ratios—indices of sustained attention—were ~15% smaller and predicted faster correct detections across individuals, confirming that midline oscillatory balance mirrors real-time cognitive control [45].

A similar one-to-one scaling appeared in a judgment task: while scoring unfamiliar kata videos, Italian national judges reproduced the coach’s ratings with a median *r* = 0.40, and the extent of their high-alpha ERD suppression over dorsal visuospatial and mirror circuits tracked that concordance (smaller ERD ⇒ higher accuracy) across all three skill levels [51].

Postural studies extend the pattern to sensorimotor domains. In quiet upright stance, elite karateka who generated stronger high-alpha ERD at right parietal sites (CP6/C6) achieved the greatest visual-driven reduction in sway area (|*r*| = 0.61, *p* = 0.008), indicating that moment-to-moment desynchronization in multisensory cortex indexes how effectively visual cues tighten balance control [56]. Conversely, the same athletes exhibited lower alpha EEG-EMG coherence with the gastrocnemius than non-athletes, yet maintained high cortex→muscle transfer entropy, implying a leaner, more selective descending command that achieves equal postural stability with less redundant coupling [57].

Stress physiology provides a biochemical bridge. Immediately after K-1 bouts, cortisol surged from ≈313→570 nmol L^−1^ while the testosterone-to-cortisol ratio halved; the fall in this ratio predicted frontal SMR and β_1_ power gains (Kendall τ ≈ 0.33), linking endocrine load to fast-band cortical arousal [61]. In the same cohort, delta and β_2_ increases (Δ + 6.1 and + 4.8 µV) scaled linearly with the count of head blows filmed during the fight (ρ ≈ 0.55), demonstrating a graded electrophysiological imprint of mechanical impact [60]. Even before combat begins, pre-fight recordings show frontal theta and β_2_ at the upper normative limit, and both bands climb further when athletes close their eyes to visualize tactics—an anticipatory state echoing the subjective reports of internal rehearsal and rising sympathetic tone [59].

Taken together, these findings show that EEG–behavior coupling is strongest when the neural measurement is locked to the task or stressor of interest: oscillatory ratios capture attentional fidelity, alpha ERD gauges judgment precision and balance gains, coherence patterns reveal economical muscle drive, and fast-band power shifts mirror both hormonal surges and the immediate biomechanical burden of blows. Such situational metrics therefore hold greater promise for performance monitoring and acute injury assessment than resting-state markers alone.

## 5. Limitations and Future Directions

The studies included in this review differed significantly in terms of methodology, participant characteristics, EEG measurement, analytical approaches, and other parameters. This heterogeneity causes some difficulties in the comparability and reproducibility of results. Below, we outline the most important limitations and suggest directions for future research.

### 5.1. Methodological Heterogeneity

Across the 23 combat sport studies, technical, analytical, and statistical approaches spanned almost the full breadth of modern EEG, making cross-study synthesis precarious.

Channel count ranged from a single Cz derivation for theta/beta ratios in amateurs [45] to 64-lead high-density caps used for graph-theoretical analysis [44]. However, the International Federation of Clinical Neurophysiology (IFCN) now recommends at least 21 electrodes, ≥16-bit A/D resolution, and ≤1 µV RMS noise for research-grade EEG [85]. Only two of the 23 studies reported amplifier resolution, and none reported noise levels. Reference montages also varied—linked ears, average, REST, and surface Laplacian; each can alter spectral power estimates and invert network-hub rankings [86].

Another methodological limitation was the defined range of EEG frequency bands. Studies differed in their definition of activity, e.g., alpha, which resulted in limited comparability. Furthermore, some studies divide frequency bands into sub-bands. This introduces additional heterogeneity that complicates comparisons between results. Future studies should align with established frequency definitions to enhance interpretability and facilitate meta-analysis.

### 5.2. Sample Composition and Exposure Bias

The EEG data across the 23 studies came from samples that do not represent the broader combat sport population. More than 85% of participants were men. Women were included in only three mixed-sex karate or fencing cohorts and made up just a quarter of those samples. This matters as female athletes are known to experience more severe and longer-lasting post-concussive symptoms [87,88,89,90] and are at higher risk for repeat concussion even after clinical clearance [91]. Ignoring sex-based differences risks encoding male-centric norms into future EEG screening tools and could delay diagnosis in female fighters.

Age was another blind spot. About one-third of the included studies recruited adolescents or collegiate amateurs, while the remainder focused on adults with over 10 years of experience. However, longitudinal MEG research in school-age American football players has shown that a single season of subconcussive impacts is enough to drive a measurable, global rise in resting delta power [92]. Combining youth and adult data without age adjustment obscures the developmental trajectory of electrophysiological change and makes it impossible to separate age-related brain maturation from trauma-induced changes.

Selection bias compounds these issues. Nearly all cohorts were composed of volunteers recruited from clubs, national teams, or rehabilitation clinics. Athletes who were either concerned about their health (or, conversely, those who felt completely healthy) were more likely to participate, skewing results. Some studies also reported lower enrollment among fighters with poor competitive records or recent knockouts, again biasing the samples toward the healthier individuals. Volunteer bias is a well-known problem in concussion research, where motivations linked to athletic identity or scholarship eligibility influence willingness to report symptoms or attend laboratory sessions [93].

Finally, the comparison groups themselves were heterogeneous. Some papers matched combat athletes to sedentary peers, others to ball sport players who accumulate their own subconcussive impacts, and six studies dispensed with live controls altogether, benchmarking EEGs against historical laboratory norms collected on outdated amplifiers. Such between-study differences in control selection alone can shift the Z-score boundary that defines “abnormal” by up to one full standard deviation, eroding external validity.

### 5.3. Lack of Longitudinal, Multimodal Evidence

Despite four decades of work, the EEG literature on combat sports remains almost entirely cross-sectional. None of the 23 reviewed papers followed athletes over an entire season, and only 2 repeated EEG within a 1 h window (the judo choke study and the kickboxing fight study). Without true baseline and follow-up data, it is impossible to know whether the spectral slowing, alpha-reactivity blunting, or network-topology erosion reported in boxers and kickboxers represented (i) pre-existing inter-individual variability, (ii) short-lived physiological stress, or (iii) a progressive neurodegenerative trajectory.

Another limitation was the narrow focus on EEG as a standalone tool. Only three studies acquired any measure beyond EEG (one used paired rCBF xenon-133, two recorded salivary or serum hormones), and none combined EEG with modern fluid biomarkers such as neurofilament light or phosphorylated tau, which are sensitive to axonal and microtubular injury in sport-related concussion cohorts. No studies combined EEG with arterial spin labeling perfusion MRI, tau-PET, or MEG, even though reviews now rank multimodal designs as the most promising route to objective staging of brain recovery after mild TBI [94,95,96].

Future studies should embed EEG into large-scale longitudinal platforms (e.g., PFBHS, NCAA CARE, military blast registries) and adopt a multimodal core battery—resting and task EEG/MEG, 3 T MRI with DTI/ASL, serum NfL/t-tau, plus head impact telemetry—so that converging evidence can isolate vascular, axonal, metabolic, and network drivers of dysfunction, schedule repeated sampling at clinically meaningful inflection points (pre-season, mid-season, post-knockout, and post-off-season) to separate transient neurometabolic “spikes” from cumulative drift, and leverage portable dry-sensor headsets to capture high-density EEG ringside and during training, minimizing loss to follow-up and enabling dense individual growth-curve modeling. Until such designs are implemented, any claim that combat sport EEG abnormalities predict long-term cognitive or neuropsychiatric outcome remains speculative.

### 5.4. Relating Other Functional Outcomes of Athletes to EEG Outcomes

It is well established that combat sport athletes are at risk of head injuries and associated cognitive decline and deficits [97,98]. Future studies should examine how cognitive performance relates to EEG abnormalities, with attention to specific domains, such as attention or working memory. EEG may be a useful tool because this type of neuroimaging can track cognitive declines in healthy and sick individuals [99,100,101,102].

Another relevant variable is education. Higher education has been shown to protect professional fighters against cognitive decline [103]. It would be useful to assess whether EEG patterns differ by educational attainment and whether this variable moderates vulnerability to trauma-related EEG changes.

### 5.5. Translational and Regulatory Gaps

Despite the growing body of laboratory evidence linking combat sport participation to reproducible EEG signatures, almost none of those metrics have crossed the threshold into day-to-day medical supervision, return-to-play (RTP) decisions, or licensing. Current concussion and combat sport regulations still rely almost exclusively on symptom check-lists, time-based lay-offs, and, at best, structural imaging.

First, clinical guidelines continue to classify quantitative EEG as investigational. For example, the 2021 American Clinical Neurophysiology Society practice guidelines concluded that the existing evidence “does not support the clinical use of qEEG either at the time of injury or remote from injury to diagnose mTBI” and assigned the technique Class III/Level U status [104]. As a result, EEG-based biomarkers are rarely reimbursed by insurers and remain underutilized.

Second, combat sport governing bodies mandate structural imaging but ignore functional electrophysiology. The Association of Ringside Physicians, for example, recommends MRI in some cases, but does not include EEG or qEEG in its licensing or post-knockout clearance guidelines [105].

Third, there is no agreed-upon analytical or legal standard for a “clinically significant” EEG deviation in an otherwise asymptomatic fighter. Normative databases (e.g., NeuroGuide, BrainDx) are composed largely of sedentary controls; applying their Z-scores to athletes whose resting rhythms are shaped by years of intense training risks high false-positive or false-negative rates.

Finally, practical and ethical barriers slow adoption. Portable 32–64-channel dry-electrode systems can now record artifact-free data in under 5 min, yet the ringside environment still poses challenges: sweat, movement, and time pressure can compromise signal quality. Data storage raises privacy concerns, and implementing advanced analytics demands neuro-informatics expertise rarely available to team physicians. Until organizations fund the necessary hardware, training, and secure infrastructure, integration into routine care will remain limited.

In summary, the regulatory lag is not due to a lack of promising science but to five intersecting problems: (i) guideline skepticism driven by heterogeneous evidence, (ii) combat sport rules that privilege structural over functional tests, (iii) limited sport-specific validation of commercial EEG devices, (iv) the absence of biomarker qualification pathways and normative athlete databases, and (v) logistical constraints. Closing these gaps will require multicenter prospective studies that align with regulatory science frameworks and negotiate with governing bodies to embed EEG into routine medical oversight.

### 5.6. Taking State Confounders into Account

State confounders can invert or camouflage genuine trauma signatures: an over-aroused, sleep-deprived, mildly dehydrated but otherwise healthy athlete may display the same frontal beta-2 elevation and parietal alpha suppression as a chronically concussed peer. Robust interpretation therefore demands multimodal state monitoring—heart rate variability for arousal, urine osmolality for hydration, actigraphy for sleep, salivary cortisol for endocrine load, and head-motion sensors for artifactual beta. Incorporating these variables does not weaken the value of EEG; it clarifies its meaning and helps distinguish between red flags that require medical follow-up and benign fluctuations linked to training conditions.

### 5.7. Under-Explored Research Questions

Despite four decades of research, several strategically important questions remain largely unanswered.

The first concerns sex-specific neurophysiology. Only about 10% of the total sample across the 23 reviewed studies were women, and none of the EEG papers stratified their findings by menstrual phase, oral-contraceptive use, or hormonal profile. As mentioned earlier, female athletes experience higher rates of sport-related concussion than males, recover more slowly, and show different neuroendocrine responses. Future multimodal concussion and TBI studies in combat sports may reveal sex-linked EEG patterns—for example, greater post-impact frontal theta or slower alpha recovery—but as of now, no such data exist in the context of combat sports.

The second question relates to developmental trajectories in youth athletes. No longitudinal EEG study has tracked adolescents as they progress to senior levels. Pediatric concussion research shows that slow-wave activity and alpha-band connectivity diminish with brain maturation but re-emerge after repeated head impacts—especially if those impacts occur before age 15 [106]. High-density EEG collected annually through puberty, along with head impact logs, are needed to map how exposure interacts with development and to establish “safe” sparring thresholds for minors.

The third is the potential role of neuromodulatory interventions. Randomized trials of head-gear redesign, mouthguard sensors, or cervical strength training have not included EEG end-points. Likewise, no study has evaluated whether non-invasive brain stimulation can accelerate post-bout EEG normalization, despite promising dual-mode tDCS protocols that enhanced visuo-motor acuity in taekwondo athletes [107]. Future research should investigate whether neurostimulation or EEG-based neurofeedback can normalize altered brain activity in combat sport athletes. The fundamental question is whether neurotherapeutic techniques can reverse the trauma-related EEG abnormalities and restore a more balanced state of cortical function.

## 6. Conclusions

EEG studies in combat sport athletes expose two parallel stories: (i) early injury signals—such as lower alpha/theta power, slow-wave increases, and disrupted small-world networks—that may foreshadow structural damage and (ii) training-driven “neural efficiency”, where elites show higher resting alpha, smaller task-related desynchronization, and leaner cortico-muscular coupling. Acute stress adds a third layer: real bouts briefly spike delta and high-beta power in proportion to head-blow count and cortisol, whereas vascular occlusions from chokes trigger only short-lived slow-wave bursts.

The literature, however, is patchy: electrode montages, band definitions, and artifact controls vary, most studies are cross-sectional and male, and multimodal data are rare. Standardized 21-plus-channel protocols, harmonized analysis bands, and longitudinal cohorts that pair EEG with impact telemetry, blood biomarkers, MRI, and cognition are now the critical next steps.

With emerging dry-sensor headsets, ringside EEG could soon inform concussion triage, return to play, and targeted interventions such as neurofeedback or cervical strength training—provided governing bodies adopt uniform metrics and insurers acknowledge their value. Properly standardized, EEG offers a fast, inexpensive window on both risk and resilience in combat sports and could become a cornerstone of athlete brain health.

## Data Availability

No new data were created or analyzed in this study. Data sharing is not applicable to this article.
